# Laser Preset of MnO_x_ Layer on High‐Entropy Alloy Surface for Ampere‐Level Ultra‐Stable OER Performance

**DOI:** 10.1002/advs.76313

**Published:** 2026-06-30

**Authors:** Benzhi Wang, Ziyang Duan, Jeong Yeon Heo, Thu Ha Le, Byunggon Song, Sunhyeong Kwon, Ji Hoon Lee, Jonghwan Suhr, Hyung Mo Jeong

**Affiliations:** ^1^ Institute of Advanced Machinery and Technology (IAMT) Sungkyunkwan University Suwon Republic of Korea; ^2^ School of Mechanical Engineering Sungkyunkwan University Suwon Republic of Korea; ^3^ School of Materials Science and Engineering and KNU Advanced Material Research Institute Kyungpook National University Daegu Republic of Korea; ^4^ Department of Smart Fab. Technology Sungkyunkwan University Suwon Republic of Korea

**Keywords:** high‐entropy alloy, industrial‐grade stability, laser powder bed fusion, oxygen evolution reaction

## Abstract

Electrocatalytic water splitting offers an efficient pathway for producing renewable hydrogen, which is considered a key strategy toward global carbon neutrality. Yet, under harsh industrial operating conditions, the catalyst inevitably undergoes dynamic structural reconstruction, oxidative dissolution of active sites, and mechanical detachment during high‐potential anodic oxygen evolution reaction (OER), severely limiting their activity and stability. Here, we present an MnO_x_ (composed of Mn_3_O_4_ and Mn_2_O_3_) layer on the FeCoNiCrMn high‐entropy alloy (HEA) surface (denoted as HEA‐ML) via laser powder bed fusion (LPBF) process and reveal that the MnO_x_ layer suppresses multimetal dissolution in HEA and mitigates excessive surface reconstruction under OER conditions. Furthermore, the MnO_x_ layer featuring a porous and rough surface enhances water adsorption and bubble diffusion, thereby accelerating interfacial mass transfer and OER kinetics. These outstanding features endow the catalyst to exhibit both excellent activity (227 ± 2 mV at 10 mA cm^−2^) and industrial‐grade ultra‐stable OER performance (operating stably for 1500 h at 1.0 A cm^−2^). Our research findings provide new insights into the structure‐activity‐stability relationship of HEA electrocatalysts and demonstrate a novel approach for designing industrially viable OER catalysts with high activity and ultra‐stability.

## Graphical abstract

3D printing enables a high entropy alloy‐derived, in situ formed MnO_x_ overlayer that suppresses oxidation–reconstruction and mitigates anodic dissolution, while enhancing electrolyte wetting and bubble release to accelerate interfacial mass transport and OER kinetics. The integrated architecture delivers industrial‐grade durability, operating stably for more than 1500 h at ultrahigh current densities.

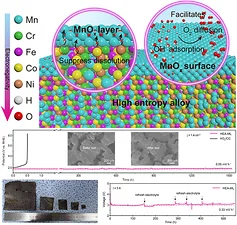

## 1 Introduction

The development of renewable energy through electrochemical water splitting plays a crucial role in alleviating the energy crisis and achieving global carbon neutrality goals [1, 2]. However, the sluggish reaction kinetics of the oxygen evolution reaction (OER) at the anode significantly limit the performance and industrial application prospects of electrochemical water splitting [3, 4, 5]. Consequently, significant efforts have been focused on developing efficient, cost‐effective anode catalysts that meet industrial requirements. Despite considerable advancements made by researchers in addressing these challenges, it remains difficult to fully meet the industrial requirements for OER catalysts, such as low cost, high activity, long‐term durability, and simplified manufacturing processes [6, 7, 8]. Particularly under high current density (≥ 500 mA cm^−2^) conditions, catalysts inevitably suffer from oxidative dissolution and fall off from the substrate [9, 10]. In recent years, the construction of robust surface oxide layers has emerged as a highly effective strategy to mitigate catalyst deactivation under harsh oxidative conditions [11, 12]. These surface oxide layers not only suppress metal leaching and facilitate charge and ion transport but also act as electrochemical protection barriers to enhance durability [13]. For example, Wang et al. demonstrated an epitaxially grown Ni(OH)_2_ interfacial layer that serves as an electronic bridge between FeCo‐LDH active species and a Ni foam substrate, simultaneously enhancing both catalytic activity and stability [14]. Similarly, Khamene et al. employed plasma‐assisted atomic layer deposition (ALD) to fabricate NiO coatings, which significantly improved OER performance [15]. Despite considerable progress in developing surface oxide layers, they often suffer from poor lattice matching with the substrate and limited interfacial adhesion, which can result in cracking or delamination under high anodic potentials during OER [16, 17, 18], ultimately constraining the catalyst's potential for industrial application.

High‐entropy alloys (HEAs), characterized by their multi‐element composition and significant electronegativity differences, which can induce the in situ generation of multi‐element gradient oxide layers, offering unique benefits for constructing chemically compatible and mechanically resilient surface oxide layers [19, 20, 21]. Specifically, when manganese with reversible multivalent states is incorporated into HEA, the in situ‐formed manganese‐based oxide imparts excellent redox buffering capability, which dynamically stabilizes the surface under highly oxidative conditions, thereby suppressing over‐oxidation and metal dissolution [22]. In addition, its abundant oxygen vacancies and mixed conductivity facilitate interfacial charge transfer and enhance the adsorption and conversion of reaction intermediates [23, 24]. Importantly, manganese‐based oxide exhibits self‐healing and reconstruction behaviors during operation [25, 26], helping to maintain the overall structural stability of the catalyst and enabling both high activity and long‐term durability. However, the fabrication methods currently employed are difficult to scale up to industrial levels. Therefore, it remains to be demonstrated whether manganese‐based oxide catalysts can be in situ grown through a readily scalable strategy while simultaneously exhibiting the aforementioned superior properties. At present, although solvothermal and hydrothermal methods can construct core–shell or heterostructure for constructing oxide layers, they are complex and suffer from poor reproducibility [27, 28]. In contrast, simple coating or atomic layer deposition is facile but often produces weakly adhered layers, limiting long‐term stability under high current densities [29, 30]. Recently, laser‐based 3D printing has emerged as a potential technique for synthesizing advanced core–shell structures by exploiting the strong reactivity between the molten pool and the chamber atmosphere during the laser powder bed fusion (LPBF) process. Specifically, the laser rapidly heats the metallic powders to momentarily reach ∼3000°C, enabling instantaneous in situ reactions between oxygen and the molten metal at the surface [31]. This process is highly flexible and efficient, as it leverages the built‐in gas control systems of most printers and eliminates the additional fabrication steps.

Here, we propose the concept of a laser preset oxide layer, in which MnO_x_—primarily composed of Mn_3_O_4_ and Mn_2_O_3_—is rapidly in situ formed on the Cantor HEA (FeCoNiCrMn) surface (denoted as HEA‐ML) through the LPBF process to achieve high OER catalytic activity and ultra‐long stability under industrial conditions. Specifically, when FeCoNiCrMn HEA is processed in an air atmosphere via the LPBF. Owing to the most negative oxidation free energy of Mn among all constituent elements, oxygen preferentially reacts with Mn during printing under oxidative conditions, leading to the spontaneous in situ formation of an MnO_x_ layer on HEA surface [32, 33]. The MnO_x_ layer preset on the HEA surface can not only shorten the oxidation process of the HEA during OER but also facilitate the diffusion of OH^−^. This accelerates the catalyst's kinetic processes, resulting in high catalytic activity (Figure 1a). Furthermore, the MnO_x_ acts as a stabilizing protective layer that suppresses multimetal dissolution in the high‐entropy alloy during the OER process, thereby mitigating excessive surface reconstruction and enabling exceptional robustness. This surface preset strategy paves the way for catalysts to achieve both excellent industrial‐grade catalytic activity and long‐term stability under harsh oxidative conditions.

**FIGURE 1:**
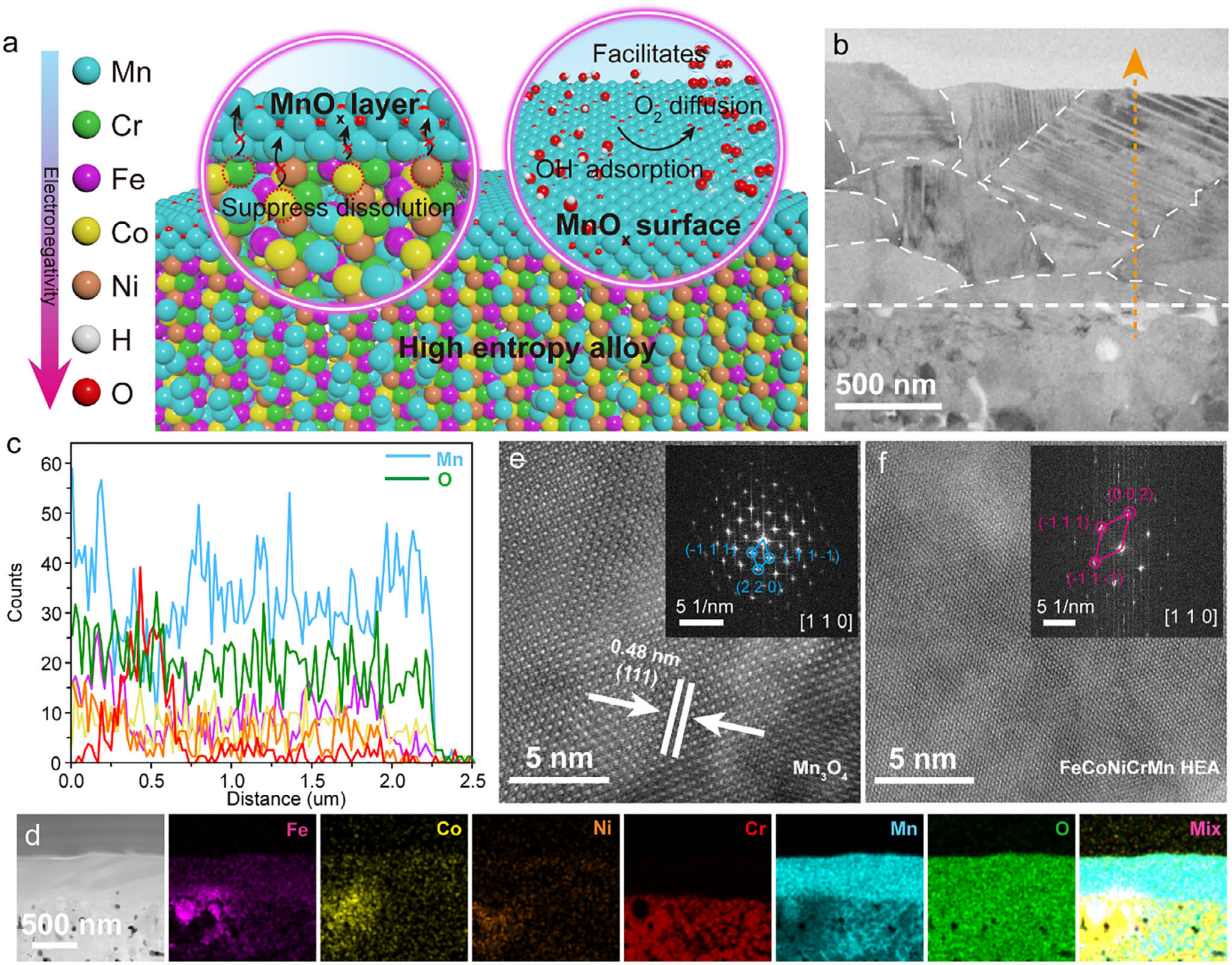
(a) Schematic diagram of a preset manganese oxide layer on the HEA surface by LPBF to accelerate OER kinetics and robustness. (b) TEM image and line scan profile of the HEA‐ML. (c) EDS line scan profiles of HEA‐ML along the marked line. (d) EDS elemental mapping images of HEA‐ML. (e, f) HAADF‐STEM images and the corresponding fast Fourier transform (FFT) image of HEA‐ML.

## 2 Results and Discussion

### 2.1 Formation of MnO_x_ Layered on HEA Surface

The catalysts were synthesized using a LPBF process under nitrogen protection and air conditions and were named HEA and HEA‐ML, respectively (Figure S1). First, cross‐sectional scanning electron microscopy (SEM) elemental line scan profiles were performed on HEA and HEA‐ML, and the results showed that the elements were uniformly distributed on the cross‐section of HEA. In contrast, HEA‐ML showed a concentrated spectra signal distribution of manganese and oxygen elements (Figure S2). This proves that a manganese oxide layer was formed on the surface of the HEA under oxygen induction. Transmission electron microscopy (TEM) analysis was conducted to further confirm the presence of a manganese oxide layer in HEA‐ML. TEM samples were prepared using a focused ion beam/SEM (FIB/SEM), as displayed in Figure S3. The TEM images revealed that HEA‐ML consists of two layers, with the surface layer forming larger grains (Figure 1b). The energy‐dispersive X‐ray spectroscopy (EDS) line scan profiles exhibit significantly higher Mn and O element content on the surface, as shown in Figure 1c. Further investigation was carried out by EDS mapping (Figure 1d). The results confirmed that a manganese oxide layer with an estimated thickness of approximately 500 nm formed on the HEA surface when printed under air conditions, whereas a manganese oxide layer did not form under nitrogen conditions (Figure S4). The atomic‐scale high‐angle annular dark‐field scanning transmission electron microscopy (HAADF‐STEM) images and the corresponding fast Fourier transform (FFT) confirm that the surface has a cubic Mn_3_O_4_ crystallographic configuration, as displayed in Figure 1e. The cubic crystal configuration of the substrate HEA was also demonstrated through HAADF‐STEM and the corresponding FFT in Figure 1f. The microstructural characterization (SEM, TEM, EDS mapping, and HAAD‐STEM) demonstrates that the laser induces the exsolution of Mn from the high‐entropy alloy matrix, which subsequently reacts with oxygen to form a manganese oxide layer on the surface.

The X‐ray diffraction (XRD) confirmed that the diffraction peaks of the HEA were consistent with a typical cubic $\left(\mathrm{Fm}\bar{3}m\right)$ Cantor alloy (Figure 2a) [34]. Notably, in addition to exhibiting the characteristic diffraction peaks of the Cantor alloy, HEA‐ML also displayed the characteristic diffraction peaks of cubic $Mn_{2}O_{3}\;\left(\mathrm{Ia}\bar{3}\right)$) and cubic Mn_3_O_4_ $\left(\mathrm{Fd}\bar{3}m\right)$ [35]. This proved the successful formation an MnO_x_ layer, mainly composed of Mn_3_O_4_ and Mn_2_O_3_ in HEA‐ML, consistent with the HAAD‐STEM results. Moreover, as displayed in Figure 2b, the Raman spectra of HEA‐ML exhibited strong vibration peaks at 310, 363, and 651 cm^−1^, which were attributed to the bending vibration and symmetric vibration V_2_ of the Mn─O [36], respectively. The X‐ray photoelectron spectroscopy (XPS) further investigated the surface of HEA‐ML. The XPS spectra of binding energy were calibrated using C 1s as a reference (Figure S5a). Subsequently, the XPS survey and high‐resolution spectra of the catalyst were analyzed. The XPS survey spectra revealed the presence of Cr, Mn, Fe, Co, Ni, and O in HEA‐ML, with Mn and O exhibiting significantly higher concentrations than the other elements (Figure S5b). This was attributed to the formation of a manganese oxide layer on the surface, corresponding to the EDS mapping result. The high‐resolution Mn 2p spectra of HEA and HEA‐ML are shown in Figure 2c. The peak around 639 eV was typically attributed to metallic Mn [37]. Compared to HEA, HEA‐ML did not exhibit the characteristic peak of metallic Mn, which was attributed to the oxidation of manganese on the surface.

**FIGURE 2:**
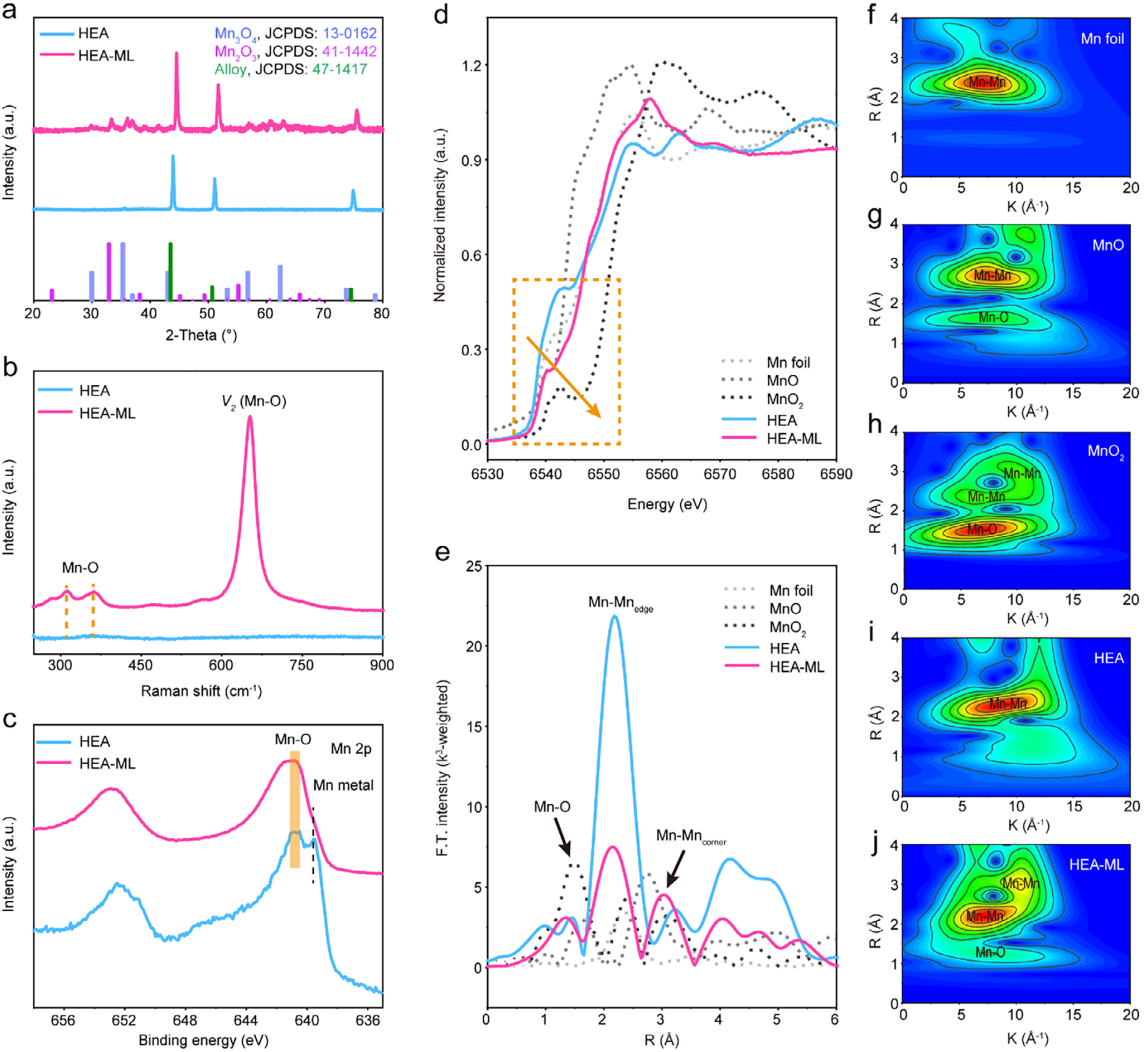
Chemical structure characterization of the preset MnO_x_ layer. (a) XRD patterns of HEA and HEA‐ML. (b) Raman spectra of HEA and HEA‐ML. (c) The high‐resolution Mn 2p XPS of HEA and HEA‐ML. (d) Mn K‐edge XANES spectra and (f) Fourier transform (FT)‐EXAFS spectra of Mn foil, MnO, MnO_2_, HEA, and HEA‐ML. (f–j) wavelet transform (WT)‐EXAFS spectra of Mn foil, MnO, MnO_2_, HEA, and HEA‐ML, respectively.

To understand the relative oxidation states and coordination environments of Mn in HEA and HEA‐ML, an X‐ray absorption fine structure (XAFS) study was conducted. Figure 2d shows the Mn K‐edge X‐ray absorption near‐edge structure (XANES) spectra of Mn foil, MnO, MnO_2_, HEA, and HEA‐ML. All Mn compounds exhibited a pre‐edge absorption feature around 6540 eV, corresponding to the transition of electrons from the occupied 1s orbital to partially unoccupied 3d orbitals [38]. The HEA exhibited a pre‐edge absorption feature similar to that of Mn foil, confirming the metallic nature of Mn in HEA. In contrast, the absorption edge of the Mn K‐edge in HEA‐ML was located between those of Mn foil and MnO_2_, indicating that Mn was oxidized with a valence state between 0 and 4^+^ [39]. To further determine the average valence state of Mn in HEA‐ML, a dependency relationship was established based on the edge energy of Mn foil, MnO, and MnO_2_ concerning the Mn formal valence (0 to 4^+^). As a result, the valence state of Mn in HEA‐ML was determined to be 2.6^+^ (Figure S6). The extended X‐ray absorption fine structure (EXAFS) spectra of Mn foil, MnO, MnO_2_, HEA, and HEA‐ML were further analyzed. The Fourier transform (FT) k^3^‐weighted EXAFS of HEA‐ML exhibited three main peaks, as shown in Figure 2e. The peak located at 1.36 Å corresponded to the first‐shell Mn─O bond, while the characteristic peaks at 2.13 and 3.04 Å corresponded to the second‐shell Mn─Mn edge‐sharing (Mn─Mn_edge_) and the third‐shell Mn─Mn corner‐sharing (Mn─Mn_corner_) [40], respectively. Furthermore, wavelet transform EXAFS (WT‐EXAFS) provides high resolution in both R and K space, enabling the detailed observation of the located structure in the composite material. In Figure 2f–j, WT‐EXAFS analysis was performed on Mn foil, MnO, MnO_2_, HEA, and HEA‐ML to verify the presence of MnO_x_ in HEA‐ML. The higher intensity maximum of Mn foil was located at 7.3 Å^−1^, attributed to the backscattering of metallic Mn─Mn coordination (Figure 2f). For MnO_2_, three distinct intensity maxima were observed at 6.8, 6.3, and 10.0 Å^−1^, corresponding to the Mn─O, Mn─Mn_edge_, and Mn─Mn_corner_ scattering paths (Figure 2h), respectively. Similarly, these three intensity maxima were also detected in HEA‐ML (Figure 2j), confirming the presence of a manganese oxide layer in HEA‐ML. Comprehensive characterization HAAD‐STEM, XRD, Raman, XPS, and XAFS consistently confirms that the LPBF process successfully induces the in situ growth of a MnO_x_ (composed of Mn_3_O_4_ and Mn_2_O_3_) layer on the HEA surface. This in situ–grown MnO_x_ layer structure enables high OER activity and robust stability, particularly under high current‐density operation.

### 2.2 Electrocatalytic OER Performance of HEA Electrodes

The catalytic performance of these catalysts was evaluated using a three‐electrode configuration in 1.0 M KOH electrolyte at room temperature with 85% iR compensation. As shown in the linear sweep voltammetry (LSV) curves of HEA, HEA‐ML, and commercial iridium oxide on carbon cloth (IrO_2_/CC) in Figure 3a. The HEA‐ML exhibits superior electrocatalytic activity compared to HEA and IrO_2_/CC. Specifically, HEA‐ML only requires an ultra‐low overpotential of 227 ± 2 mV to achieve a geometric current density of 10 mA cm^−2^, which is 84 and 66 mV lower than those of HEA (311 ± 4 mV) and IrO_2_/CC (293 mV), respectively. This pronounced reduction of overpotential can be attributed to the formation of MnO_x_ on the HEA surface. In addition, HEA‐ML also exhibited competitive OER performance compared to previously reported HEA‐based catalysts (Table S1). Typically, the kinetics of OER can be evaluated using the Tafel slope. As shown in Figure 3b, HEA‐ML exhibited a lower Tafel slope of 39.4 mV dec^−1^ compared to HEA (47.9 mV dec^−1^) and IrO_2_ (63.5 mV dec^−1^), indicating a faster reaction kinetics process [41, 42]. When considering the simultaneous achievement of low overpotential (η_10_) and small Tafel slope in the catalyst, HEA‐ML also exhibited a leading performance compared to previously reported HEA‐based catalysts (Figure S7 and Table S1). Furthermore, the electrochemically active surface area (ECSA) was measured, and the geometric current density was normalized to the corresponding ECSA to estimate the intrinsic activity of the catalysts [43]. The results demonstrated that HEA‐ML exhibited superior intrinsic activity compared to HEA and IrO_2_/CC (Figure 3c; Figures S8 and S9). To evaluate the stability of the HEA‐ML, cyclic voltammetry (CV) testing was first conducted. After 50 000 CV cycles, the HEA‐ML exhibited no significant performance degradation (Figure 3d and Figure S10). The catalyst stability at high current densities (≥500 mA cm^−2^, up to ampere‐level) was considered a critical benchmark for industrial water electrolysis [44]. However, under such conditions, the catalyst faces extreme polarization, bubble accumulation, and mechanical stress, and also undergoes oxidative dissolution, resulting in the loss of active metal sites and structural degradation, which compromise long‐term stability [45]. To realistically assess durability under such conditions, the catalyst was evaluated using a chronoamperometry (CP) method at 1.0 A cm^−2^, offering insights into its high‐load performance and structural stability. As shown in Figure 3e, the stability of IrO_2_/CC was significantly poorer, failing after only about 0.5 h. This degradation was primarily attributed to catalyst detachment from the substrate and dissolution at high current density, resulting in the loss of active sites—a pervasive and unavoidable issue for powder catalysts in industrial applications. Impressively, the HEA‐ML electrode maintained a nearly constant potential for over 1500 h, with a remarkably low degradation rate of 0.05 mV h^−1^. During the prolonged test, the electrolyte was intermittently replenished to minimize concentration and pH fluctuations, ensuring stable electrolyte conditions. The surface structure of the HEA‐ML electrode remained intact after the long‐term stability test lasting over 1500 h (the inset of Figure 3e). XRD analysis revealed that the diffraction peaks of Mn_2_O_3_ and Mn_3_O_4_ were still present after the extended stability test (Figure S11), indicating that the MnO_x_ layer remained stable throughout the catalytic process. In addition, inductively coupled plasma optical emission spectroscopy (ICP‐OES) analysis of the electrolyte during the stability test revealed negligible dissolution of Fe, Co, and Ni, while Mn and Cr exhibited slight leaching at the initial stage, possibly associated with the dissolution of relatively unstable surface species formed during the LPBF process, before gradually reaching a stable state (Figure S12). These results suggest that the MnO_x_ layer plays a protective role by suppressing substrate dissolution during long‐term stability tests. Notably, benefiting from the rapid prototyping and structural customizability of LPBF, electrodes of various sizes can be efficiently fabricated, enabling seamless scaling from the laboratory level to industrial dimensions. As shown in Figure 3f, HEA‐ML electrodes with different sizes were successfully prepared (1 to 100 cm^2^). This advances a viable technological pathway for the reliable operation of large‐area electrodes under high current‐density conditions. To substantiate this, a 25 cm^2^ HEA‐ML was employed as the working electrode and a graphite plate as the counter electrode to assemble a two‐electrode system (Figure S13), and a CP test was conducted at a current of 5 A. The catalyst exhibited stable operation for 550 h, with a degradation rate of only 0.33 mV h^−1^ (Figure 3g). To emphasize the industrial‐grade catalytic performance of the HEA‐ML electrode, we compared it with existing OER catalysts, including self‐supported and powder catalysts, based on three critical parameters for industrial applications: overpotential, operational voltage of stability test, and durability time. As shown in Figure 3h and Table S2, the HEA‐ML electrode not only demonstrates exceptional OER activity but also exhibits long‐term stability at a high current density of 1.0 A cm^−2^, outperforming the vast majority of state‐of‐the‐art OER catalysts. This demonstrates its unparalleled potential for industrial‐scale applications.

**FIGURE 3:**
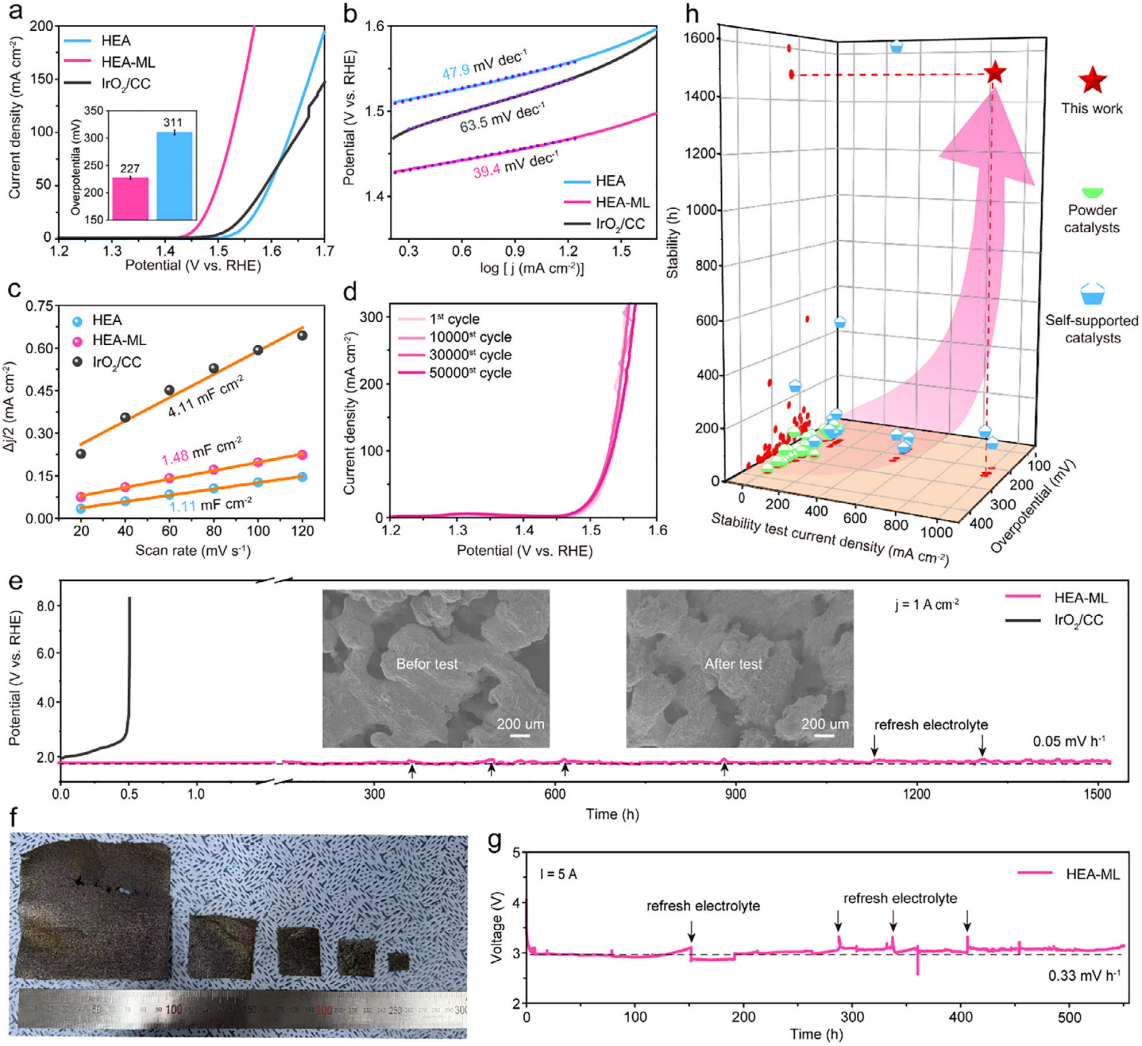
Electrocatalytic OER performance. (a) The LSV polarization curves of HEA, HEA‐ML, and IrO_2_/CC catalysts in 1.0 M KOH electrolyte. (b) Tafel plots of HEA, HEA‐ML, and IrO_2_/CC. (c) Double‐layer capacitance of HEA, HEA‐ML, and IrO_2_/CC. (d) The LSV polarization curves of the HEA‐ML electrode after 10 000, 30 000, and 50 000 cycles CV tests. (e) The chronopotentiometric curves were recorded at 1.0 A cm^−2^ for the HEA‐ML catalyst. The inset shows the scanning electron microscopy (SEM) images of the catalysts before and after long‐term tests. (f) Optical photographs of HEA‐ML electrodes with different sizes via the LPBF process. (g) The chronopotentiometric curves of the 25 cm^2^ HEA‐ML catalyst recorded at a current of 5.0 A. (h) Comparison of the activity and stability of the HEA‐ML with other OER catalysts reported in the literature.

### 2.3 The Role of MnO_x_ Layer in Enhancing OER Catalytic Activity

The next question is why the presence of the MnO_x_ layer leads to outstanding OER performance. To gain deeper insights into this, a series of in situ and finite element method (FEM) experiments were conducted on the catalyst. The in situ electrochemical Raman coupling system was employed to capture the actual catalytic species under operational voltage. As illustrated in Figure 4a and Figure S14a, HEA did not exhibit any distinct characteristic peaks at low voltages, indicating that active species were not formed. As the voltage increased, two characteristic vibration peaks appeared at 476 and 557 cm^−1^, corresponding to the *E*_g_ (δ_Ni−O_) bending vibration mode and *A*_1g_ (ν_Ni−O_) stretching vibration mode of γ‐NiOOH [46, 47], respectively, and gradually intensified with the voltage. This observation confirmed that HEA undergoes surface reconstruction during the OER process, with nickel serving as the active site for the formation of the stable catalytic species γ‐NiOOH layer. For HEA‐ML, no new vibration peaks were observed throughout the entire operating voltage range except for the Mn─O vibration peaks (Figure 4b and Figure S14b). Moreover, the intensity of the Mn─O vibration peaks decreased with the increasing potential, which is associated with the interaction between Mn sites and oxygen reaction intermediates [48]. This demonstrates that Mn serves as the catalytic active site in HEA‐ML and that the presence of MnO_x_ can bypass the traditional complicated oxide reconstruction process under OER conditions. The catalytically active site was further identified by post‐reaction XPS spectrum. As displayed in Figure S15a. The high‐resolution Ni 2p XPS spectrum revealed that metallic Ni exists in HEA‐ML but not in HEA. This was attributed to the oxidation of Ni in HEA as an active site. Meanwhile, the high‐resolution Mn 2p XPS shows that the binding energy of Mn in HEA‐ML has shifted positively relative to HEA (Figure S15b), which was due to Mn being oxidized as an active site. Additionally, the Mn K‐edge XANES spectra confirmed the presence of metallic Mn phase in HEA after the reaction, whereas HEA‐ML exhibited an oxidized Mn phase with a slight shift toward higher energy after the reaction (Figure S16). This further indicates that the in situ formed MnO_x_ layer in HEA‐ML remains stably present as a key active site, effectively modulating the electronic structure and thereby significantly enhancing the OER activity and catalytic stability.

**FIGURE 4:**
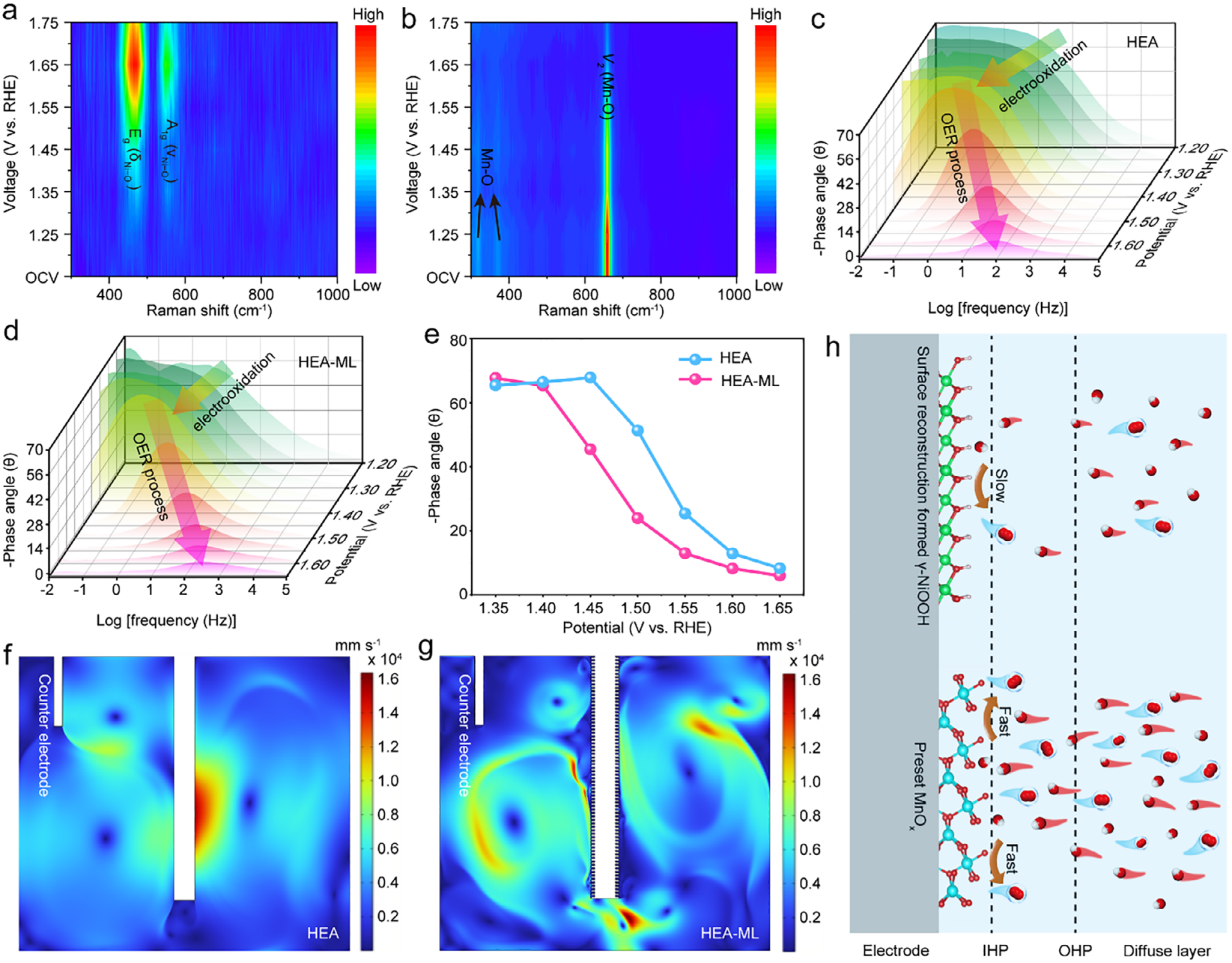
In situ investigation of the OER kinetic processes. In situ Raman spectrum of (a) HEA and (b) HEA‐ML. Bode phase plots derived from in situ EIS of (c) HEA and (d) HEA‐ML. (e) Response of the phase angle to the applied voltage for HEA and HEA‐ML. FEM simulation result of the oxygen production rate for (f) HEA and (g) HEA‐ML. (h) Schematic diagram of preset layers accelerating OH^−^ transfer and O_2_ diffusion at the electrode–electrolyte interface to promote OER.

The mass transfer process of catalysts plays a crucial role under industrial conditions [49, 50]. To investigate the influence of the MnO_x_ layer on the mass transfer process in OER, a comprehensive kinetic analysis was performed. As displayed in Figure S17, the laser scanning images showed that HEA‐ML had an arithmetic mean height value of 43.15 µm compared to HEA (16.83 µm), indicating that the surface of HEA‐ML possessed greater roughness. The contact angle experiments showed that the HEA‐ML catalyst possesses both a smaller liquid contact angle (53.6°) and a larger gas contact angle (135.9°), as displayed in Figure S18. These results indicate that HEA‐ML simultaneously exhibits excellent electrolyte wettability and pronounced aerophobicity, which together facilitate mass transfer during the OER [51]. In situ electrochemical testing offers detailed insights into the mass transfer process of the catalyst. As shown in Figure S19, the redox currents of both HEA and HEA‐ML exhibited a linear dependence on the square root of the potential scan rate in CV tests (ranging from 10 to 700 mV s^−1^). This suggests that the rate‐determining step of metal redox was controlled by mass diffusion from the electrolyte to the electrode [52, 53]. In alkaline conditions, the redox process of active sites was typically coupled with the transfer of OH^−^ ions, which was influenced by the surface state of the catalyst [54]. Additionally, in situ electrochemical impedance spectroscopy (EIS) measurements at various applied voltages were performed to further investigate the role of the MnO_x_ layer in the OER kinetics process (Figure S20). In the Bode phase plot, the phase angle peaks in the high‐frequency (10^2^–10^5^ Hz) and low‐frequency (10^−2^–10 Hz) regions correspond to the catalyst electro‐oxidation process and the charge transfer at the electrolyte–catalyst interface, respectively [55, 56]. As shown in Figure 4c,d, the voltage range in the high‐frequency region of HEA‐ML (1.20–1.35 versus reversible hydrogen electrode (*V*_RHE_)) was significantly narrower than that of HEA (1.20–1.40V_RHE_), indicating that the in situ grown MnO_x_ on the surface accelerated the electrochemical oxidation process. Furthermore, as the applied voltage increased from 1.35 to 1.65 *V*_RHE_, the low‐frequency phase angle of HEA‐ML exhibited a faster decline compared to HEA (Figure 4e), suggesting that the presence of the MnO_x_ layer facilitated electron transfer at the electrolyte–catalyst interface [57].

The hydrophilic/aerophobic and electrochemically active interfacial layer between the electrocatalyst and the electrolyte plays a crucial role in mass transfer of OER [58, 59]. Therefore, a finite element method (FEM) simulation was conducted to investigate the diffusion process of O_2_ at the electrode–electrolyte interface. Based on previous characterization data (XRD, XPS, in situ Raman, laser scanning, etc.), models of the MnO_x_ layer on HEA and the γ‐NiOOH layer on HEA were constructed. As shown in Figure 4f,g, it is evident that the HEA‐ML catalyst with a preset MnO_x_ layer is more conducive to O_2_ desorption from the electrode surface and exhibits a faster diffusion rate in the electrolyte (Figure 4g). This further confirms that the presence of MnO_x_ facilitates the mass diffusion process at the electrode–electrolyte interface, which is highly consistent with the experimental results. In‐depth kinetic analysis revealed that the preset MnO_x_ layer could suppress surface reconstruction of the catalyst, accelerating the electrochemical oxidation process. Moreover, it can facilitate the transfer of OH^−^ across the inner Helmholtz plane (IHP) and outer Helmholtz plane (OHP) at the electrode–electrolyte interface, as well as accelerating the diffusion of O_2_ bubbles at the diffusion layer. This endows HEA‐ML with rapid OER reaction kinetics, thereby achieving superior OER performance (Figure 4h).

### 2.4 Application of HEA‐ML in Water Electrolyzer Device under Industrial‐Scale Conditions

To explore the prospect of industrial application, as illustrated in Figure 5a, the HEA‐ML and Pt/C were employed as the anode and cathode in an anion exchange membrane water electrolyzer (AEMWE) with a 30% KOH electrolyte to simulate industrial water‐splitting performance. HEA‖Pt/C and ruthenium dioxide (RuO_2_) mesh ‖ Pt/C electrodes were also built for comparison. Figure 5b compares the AEMWE performance of HEA‐ML‖Pt/C, HEA‖Pt/C, and RuO_2_‖Pt/C. The cell voltage required for HEA‐ML‖Pt/C to reach current densities of 1.0, 2.0, and 3.0 A cm^−2^ was 2.12, 2.41, and 2.66 V, respectively, which were lower than those of HEA‖Pt/C (2.28, 2.50, and 2.85 V) and RuO_2_‖Pt/C (2.51, 2.86, and 3.05 V), indicating its superior water‐splitting performance. The corresponding EIS spectra (Figure 5c and Table S3) further revealed that HEA‐ML‖Pt/C exhibited a lower charge transfer resistance of 0.274 Ω cm^−2^ compared to HEA‖Pt/C (1.19 Ω cm^−2^) and RuO_2_‖Pt/C (0.705 Ω cm^−2^), demonstrating enhanced electrochemical reaction kinetics [60]. To further explore the industrial application potential of HEA‐ML‖Pt/C, its performance was also tested at elevated temperatures. As shown in Figure 5d, at 60°C, HEA‐ML‖Pt/C system demonstrated excellent AEMWE performance, requiring only 2.08, 2.43, and 2.68 V to achieve current densities of 1.0, 3.0, and 5.0 A cm^−2^, respectively. This performance was significantly superior to that at 25°C. Furthermore, compared to previously reported state‐of‐the‐art AEMWE electrodes, the HEA‐ML electrode also exhibited superior performance, as shown in Figure 5e and Table S4. To verify whether all electrons were utilized in the water electrolysis system, the Faradaic efficiency was measured using the water displacement method (Figure S21) at a high current density of 500 mA cm^−2^. The results showed that the Faradaic efficiency of O_2_ and H_2_ fluctuates around 96% during a continuous 30 min test (Figure 5f and Figure S21c), demonstrating the high‐water electrolysis efficiency of the HEA‐ML catalyst. At present, AEMWE was still at an early stage of development, with reported operating current densities typically ranging from 0.2 to 2.0 A cm^−2^ [61]. According to the targets outlined by the International Renewable Energy Agency, a current density of 2.0 A cm^−2^ was envisioned as a key performance benchmark for AEMWE systems by 2050 [62]. To align with this target, the long‐term durability of the catalysts at 2.0 A cm^−2^ was evaluated under extreme industrial conditions, including strong alkalinity (30% KOH) and high temperatures (60°C). As shown in Figure 5g, the HEA‐ML‖Pt/C system operated for 160 h with a negligible degradation rate (1.7 mV per hour). In contrast, RuO_2_‖Pt/C and HEA‖Pt/C could only sustain operation for approximately 0.5 and 10 h, respectively. These results demonstrate that HEA‐ML was well aligned with the performance targets projected for practical industrial AEMWE toward 2050.

**FIGURE 5:**
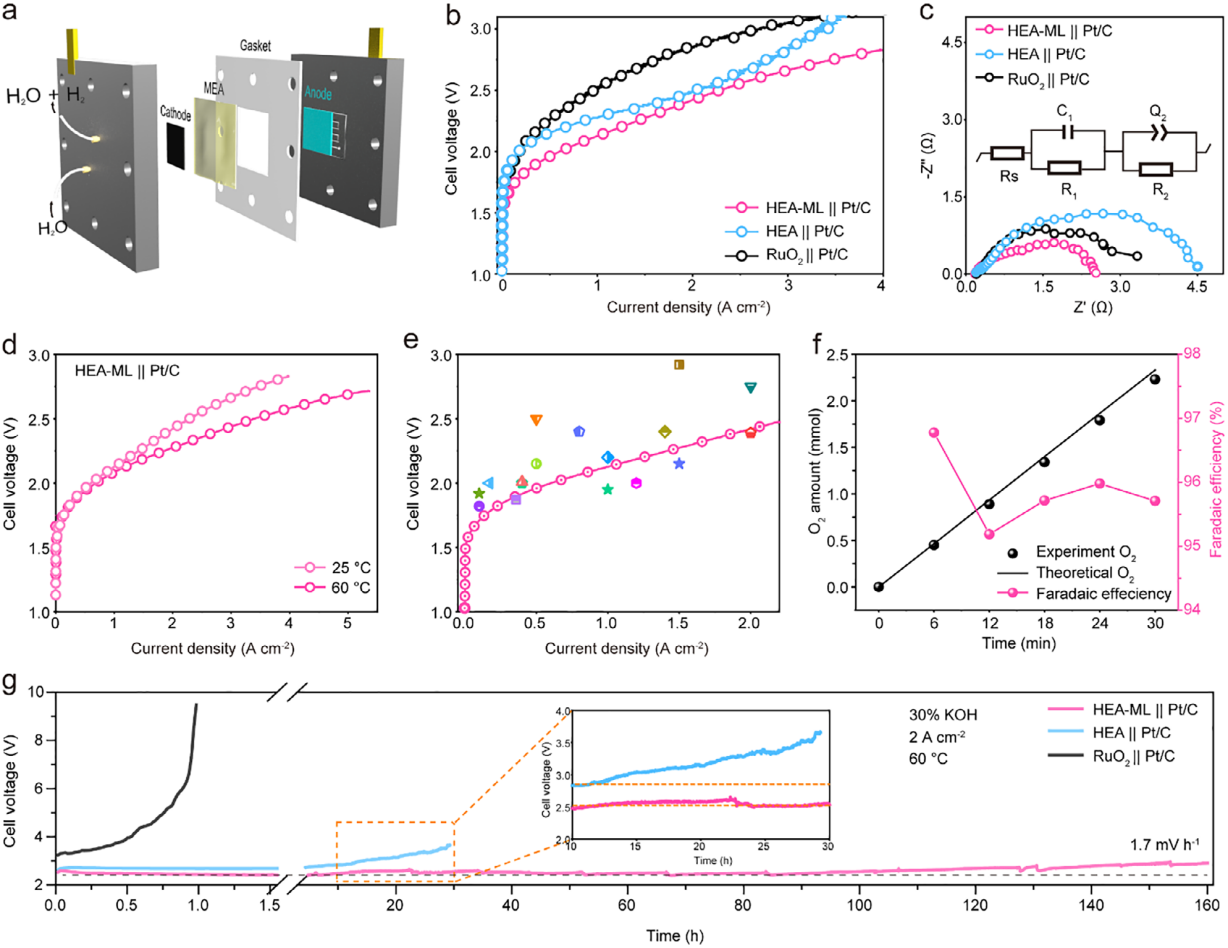
(a) Schematic diagram of the AEMWE water electrolysis device. (b) AEMWE water electrolysis performance of HEA‐ML‖Pt/C, HEA‖Pt/C, and RuO_2_‖Pt/C. (c) Nyquist impedance plots of HEA‐ML‖Pt/C, HEA‖Pt/C, and RuO_2_‖Pt/C. (d) AEMWE water electrolysis performance at different temperatures of HEA‐ML‖Pt/C. (e) Comparison of the AEMWE water electrolysis performance of HEA‐ML‖Pt/C with those reported previously electrodes. (f) Volumes of O_2_ generated and Faradaic efficiency of HEA‐ML‖Pt/C in the AEMWE water electrolysis device at 500 mA cm^−2^ under atmospheric pressure. (g) AEMWE water electrolysis stability tests of HEA‐ML‖Pt/C, HEA‖Pt/C, and RuO_2_‖Pt/C under industrial conditions.

## 3 Conclusion

In summary, we designed a novel catalyst with a unique oxide‐layer induced from FeCoNiCrMn HEA using the LPBF method. This catalyst exhibits industrial‐scale OER catalytic activity, combining outstanding activity (227 ± 2 mV at 10 mA cm^−2^) with extraordinary industrial‐level durability (>1500 h at 1.0 A cm^−2^ in alkaline electrolytes), while also ensuring scalability and cost‐effectiveness. Systematic characterization indicates that the LPBF process enables the formation of preset MnO_x_ layer on the HEA surface under oxidative conditions. The preset MnO_x_ layer effectively prevents multimetal dissolution in the HEA and mitigates excessive surface reconstruction under harsh oxidative conditions, accelerates the electrocatalytic oxidation process, and enhances mass transfer while promoting electrolyte adsorption and bubble diffusion on the catalyst surface. As a result, the catalyst exhibits rapid reaction kinetics, along with outstanding OER activity and stability. This work provides a blueprint for designing novel high‐performance catalysts that balance activity and stability, making the industrialization of the catalyst a promising prospect for the future.

## 4 Experimental Section

### 4.1 Materials

The high entropy alloy (FeCoNiCrMn) was acquired from Zhongke Yannuo (Beijing) New Material Technology Co., Ltd with an equiatomic ratio of 1:1:1:1:1. The Nafion D‐520 dispersion (5% w/w in water and 1‐propanol, >1.00 meq g^−1^ exchange capacity) was obtained from Alfa Aesar. Distilled water and absolute ethanol (C_2_H_5_OH, ≥99.7%) were procured from Daejung Chemicals. The potassium hydroxide (KOH) was sourced from Junsei. All regards, purchased from Aladdin were analytical grade and were used without further purification. Commercial IrO_2_ and Pt/C (20 wt.% for platinum) were acquired from Alfa Aesar. The RuO_2_ mesh (RuO_2_ loaded on titanium mesh with a loading of 2 mg cm^−2^) was procured from Gooss union.

### 4.2 Synthesis of HEA and HEA‐ML Porous Structure

The self‐supporting, highly active anode porous electrode was fabricated via LPBF process (SLM250 printer). The HEA powder with sizes ranging from 15 to 45 µm was spread evenly over the build surface in 60 µm‐thick layers using a powder scraper. The printing system was equipped with a continuous‐wave fiber laser (Gaussian distribution, wavelength: 1064 nm, maximum output power: 500 W). The parameters were as follows: laser power of 200 W, scanning speed of 800 mm/s, hatch spacing of 80 µm. The scanning area was 1 × 1.5 cm^2^. A double‐scanning strategy was employed, and during the second scan, the laser scanning direction was rotated by 90° relative to the first scan. The laser rapidly melts the fine powder within microseconds, followed by solidification at extremely high cooling rates, achieving values in the range of 10^5^ to 10^7^ K/s. Notably, unlike traditional printing processes, this fabrication process is carried out under nitrogen protection and ambient air to synthesize HEA and HEA‐ML, respectively.

### 4.3 Materials Characterization

Scanning electron microscopy (SEM, Quanta FEG 200, Holland), Field Emission Transmission electron microscopy at 200 kV (FE‐TEM, Talos F200X, Thermo Fisher Scientific), and Titan Double Cs corrected TEM at 300 kV (Titan cubed G2 60–300, Thermo Fisher Scientific) were used to observe material morphology and atomic structures. X‐ray powder diffraction (XRD) data were collected using a MiniFlex 600, Rigaku. The chemical states of the samples were analyzed by X‐ray photoelectron spectroscopy (XPS, K‐alpha+, Thermo Scientific). Raman spectroscopy was conducted by XperRAM‐S567. Inductively coupled plasma optical emission spectroscopy (ICP‐OES, Agilent 5900) was used to quantify the concentration of dissolved metal ions in the electrolyte. X‐ray absorption fine structure (XAFS) analyses were performed to confirm the existence of MnO_x_ in HEA‐ML at the 1D beamline (KIST‐PAL XAFS) at the Pohang Light Source (PLS‐II) in Korea. The XAFS spectra at the Mn and Ni K‐edge were obtained in transmission mode. Each spectrum was calibrated using the edge energy (*E*_0_) of the Mn and Ni foil, which was recorded simultaneously. The ATHENA and ARTEMIS software included in the IFFEFIT package were utilized to analyze the XANES and EXAFS profiles. The EXAFS spectrum (χ(k)) was weighted with the k^3^ value to enhance the signal in the high k‐regime. A Hanning window function was employed for the Fourier transformation. The wavelet transform was processed by the HAMA program.

### 4.4 Electrochemical Measurements in Three‐Electrode System

Electrochemical performance of the as‐prepared catalysts was investigated using an electrochemical workstation (VMP3, Biologic) configured with a typical three‐electrode system in 1.0 M KOH electrolyte. The printing self‐supporting electrode served as the working electrode, a graphite plate as the counter electrode, and a reversible hydrogen electrode as the reference electrode. CV tests were conducted over five cycles to stabilize the electrocatalytic performance of the catalyst at a scan rate of 20 mV s^−1^. Subsequently, LSV curves were obtained at a slow scan rate of 5 mV s^−1^, and EIS was performed near the onset potential over a frequency range from 200 kHz to 10 mHz. The Cdl was determined from the current response in the capacitive region of the CV curve at different scanning speeds in the non‐Faradaic region. All reported curves were corrected using the iR compensation.

### 4.5 Electrochemical Measurements in the AEMWE Device

The self‐printed porous HEA electrode was used as the anode catalyst, whereas commercial Pt/C (20 wt.%), deposited by spraying with a loading of approximately 1.0 mg cm^−2^, was used as the cathode catalyst. The cathode and anode catalysts were then sandwiched together with an anion exchange membrane (Dioxide Materials Sustainion37‐50) to assemble a custom‐integrated AEMWE device using a torque wrench (6 N·m). The anion exchange membrane was immersed in a 1.0 M KOH solution for at least 24 h to replace Cl^−^ with OH^−^ for the activation of the membrane. The AEMWE electrolyzer operated under ambient pressure, using 30% KOH as the electrolyte. The performance of the AEMWE was evaluated by measuring polarization curves from 1.2 to 3.0 V. The stability of the AEMWE was assessed using chronoamperometry at a current density of 2.0 A cm^−2^ at 60°C.

### 4.6 In Situ Raman Test

The in situ electrolytic cell consists of a self‐printed porous HEA as the working electrode, a graphite rod as the counter electrode, and an Hg/HgO electrode as the reference electrode. The workstation used is the same as that for the OER test. A laser with a wavelength of 532 nm was used for all Raman tests. The corresponding Raman curve is obtained by applying the chronoamperometry technique, and the electrochemical test was performed at each voltage for five minutes before data collection. Raman exposure time was 20 s, data were collected three times, and the data in the manuscript are the average data.

### 4.7 Finite Element Method Simulation

The finite element analysis used in the article is based on Comsol Multiphysics 6.1. A 2D longitudinal section of the electrolytic cell is used as a base model for the analysis, considering the accuracy of the calculations and the approximation of the described problem. The illustrative results are obtained by solving partial differential equations in electrochemistry and hydrodynamics using the quadratic current module as well as the bubble flow module due to the electrolyte concentration gradient was negligible for the simulation results. Two different materials are parametrically modeled by selecting characteristic parameters from the results of the above tests.

The tripled electrochemical and fluid flow and bubbly flow simulations are used to simulate the experimental results presented in the article. The tested hydrophilic angle, active area, and electrochemical impedance are analyzed as the main characteristic parameters. The bubble diameter parameter ratio of oxygen adsorbed on the surface was calculated from the hydrophilic angle.

Density and viscosity of the liquid phase are assumed to be uniform and are considered to be 1000 kg/m^3^ and 1.01 × 10^−3^ kg/m/s, respectively. For laminar flow, the gas velocity *u*_g_ is calculated from:

(1) $$u_{g}=u_{1}+s_{\mathrm{slip}}\;$$

where u_l_ stands for the liquid‐phase velocity, and *u*_slip_ stands for the relative velocity between gas and liquid, the so‐called slip velocity. The slip velocity is calculated from a slip model. The most appropriate slip model for this electrowinning cell is a pressure‐drag balance slip model with a drag coefficient tuned for small spherical bubbles using Hadamard–Rybczynski model. The diameter of gas bubbles is considered to be linked with the performance of hydrophilic.

The Wall boundary feature is used to set a no‐slip boundary condition for the liquid phase and to set the gas mass flux at the anode surface:

(2) $$-n\cdot N_{O_{2}}=\frac{Mw_{O_{2}}\cdot i_{loc,O}}{4F}\;$$

where is $N_{O_{2}}$ the gas mass flux for oxygen bubbles;$Mw_{O_{2}}$ is the molar mass of oxygen; *i*_loc, O_ is the local current density of oxygen evolution reaction evaluated at the quadratic current distribution, and F is Faraday's constant.

## Author Contributions

**Ji Hoon Lee**: conceptualization, formal analysis, supervision, resources, data curation, visualization, funding acquisition, writing – review and editing. **Byunggon Song**: methodology, software, investigation, validation. **Jonghwan Suhr**: data curation, supervision, resources, formal analysis, visualization, conceptualization, writing – review and editing, funding acquisition. **Jeong Yeon Heo**: methodology, software, formal analysis, investigation, visualization. **Ziyang Duan**: conceptualization, methodology, software, data curation, investigation, visualization, formal analysis, writing – original draft. **Benzhi Wang**: conceptualization, methodology, software, data curation, formal analysis, visualization, investigation, writing – original draft. **Thu Ha Le**: methodology, software, formal analysis, visualization, investigation. **Sunhyeong Kwon**: methodology, investigation, software, validation. **Hyung Mo Jeong**: conceptualization, visualization, funding acquisition, data curation, supervision, resources, project administration, writing – review and editing, formal analysis.

## Conflicts of Interest

The authors declare no conflicts of interest.

## Supporting information




**Supporting File**: advs76313‐sup‐0001‐SuppMat.docx.

## Data Availability

The data that support the findings of this study are available from the corresponding author upon reasonable request.
