# Supplementary material for: Laser Preset of MnOx Layer on High‐Entropy Alloy Surface for Ampere‐Level Ultra‐Stable OER Performance
Source: Adv Sci (Weinh). 2026 Jun 30;13(53):e76313. doi: 10.1002/advs.76313 (PMC13337119; doi:10.1002/advs.76313)
Supplement: Supplementary file 1 [file ADVS-13-e76313-s001.docx]

**Supporting Information**

**Laser Preset of MnO_x_ Layer on High-Entropy Alloy Surface for Ampere-Level Ultra-Stable OER Performance**

*Benzhi Wang^1^, Ziyang Duan^2^, Jeong Yeon Heo^3^, Thu Ha Le^4^, Byunggon Song^2^, Sunhyeong Kwon^1^,* *Byung Jun Sung^4^, Ji Hoon Lee^3,^*, Jonghwan Suhr^2^*, Hyung Mo Jeong^1,2,4,^**

^1^ Institute of Advanced Machinery and Technology (IAMT), Sungkyunkwan University, 2066 Seobu-ro, Suwon 16419, Republic of Korea

^2^ School of Mechanical Engineering, Sungkyunkwan University, 2066 Seobu-ro, Suwon 16419, Republic of Korea

^3^ School of Materials Science and Engineering and KNU Advanced Material Research Institute, Kyungpook National University, 80 Daehak-ro, Buk-gu, Daegu 41566, Republic of Korea

^4^ School of Mechanical Engineering and Department of Smart Fab. Technology, Sungkyunkwan University, 2066 Seobu-ro, Suwon 16419, Republic of Korea

*Corresponding Author

Ji Hoon Lee, jihoonlee@knu.ac.kr

Jonghwan Suhr, suhr@skku.edu

Hyung Mo Jeong, hmjeong@skku.edu

**Calculation of electrochemically active surface area (ECSA)**

The real surface area of the OER catalyst is calculated from the ECSA, and the ECSA is calculated from the specific capacitance. The specific capacitance of flat surfaces is typically in the range of 20–60 μF cm^−2^_geo_.

$$A_{ECSA}=\frac{specific capacitance}{40 \mu F {cm}_{geo}^{-2} per {cm}_{ECSA}^{-2}}$$

From Figure. 3c, ECSA can be calculated for HEA, HEA-ML and IrO_2_/CC.

$$A_{ECSA}\left( HEA \right)=\frac{1.11 mF {cm}^{-2}}{40 \mu F {cm}_{geo}^{-2} per {cm}_{ECSA}^{-2}}=27.75 {cm}_{ECSA}^{2}$$

$$A_{ECSA}\left( HEA-ML \right)=\frac{1.48 mF {cm}^{-2}}{40 \mu F {cm}_{geo}^{-2} per {cm}_{ECSA}^{-2}}=37 {cm}_{ECSA}^{2}$$

$$A_{ECSA}\left( {IrO}_{2}/CC \right)=\frac{4.11 mF {cm}^{-2}}{40 \mu F {cm}_{geo}^{-2} per {cm}_{ECSA}^{-2}}=102.75 {cm}_{ECSA}^{2}$$

**Figure S1.** Schematic illustration of the laser powder bed fusion (LPBF) synthesis of HEA and HEA-ML electrodes.

**Figure S2.** The cross-sectional SEM and corresponding line scan profile of (a) HEA and (b) HEA-ML

**Figure S3.** Cross-sectional TEM image of the HEA-ML electrode prepared by FIB

**Figure S4.** EDS elemental mapping images of HEA.

**Figure S5.** (a) High-resolution XPS of C 1s for HEA-ML. (b) XPS survey of HEA-ML.

**Figure S6.** The average oxidation states of Mn for the Mn foil, MnO, MnO_2_, HEA and HEA-ML derived from the Mn K-edge energies.

**Figure S7.** Comparison of the OER performance of HEA-ML with previously reported representative catalysts.

**Figure S8.** Cyclic voltammograms with different scan rates tested in non-faradaic potential windows in 1.0 M KOH of HEA, HEA-ML and IrO_2_/CC.

**Figure S9.** ECSA-normalized current density curves of HEA, HEA-ML and IrO_2_/CC.

**Figure S10.** CV curve plots of the stability tests of the HEA-ML electrode. All CV curve plots without iR compensation.

**Figure S11.** The XRD pattern of the before and after long time stability test for HEA-ML electrode.

**Figure S12** The concentration evolution of dissolved metal ions during the long-term stability test of the HEA-ML electrode.

**Figure S13.** Digital photographs of HEA-ML and graphite plate electrode with an area of 25 cm^2^.

**Figure S14.** The voltage-dependent in situ Raman spectra of (a) HEA and (b) HEA-ML.

**Figure S15.** (a) High-resolution XPS of Ni 2p for HEA and HEA-ML post OER. (b) High-resolution XPS of Mn 2p for HEA and HEA-ML post OER.

**Figure S16.** Mn K-edge XANES spectra of before and after OER reaction for (a) HEA and (b) HEA-ML.

**Figure S17.** Laser scanning images of HEA and HEA-ML.

**Figure S18.** (a) The wetting contact angle of HEA and HEA-ML. (b) The gas bubble contact angle of HEA and HEA-ML.

**Figure S19.** (a) CV curve plots of HEA with different scan rates in 1.0 M KOH. (b) The plot of the redox peak currents densities versus the square root of scan rates of HEA. (c) CV curve plots of HEA-ML with different scan rates in 1.0 M KOH. (d) The plot of the redox peak currents densities versus the square root of scan rates of HEA-ML.

**Figure S20.** Nyquist impedance plots at different voltages of (a) HEA and (b) HEA-ML.

**Figure S21.** Digital photographs of (a) H_2_ and (b) O_2_ gas release taken from an AEMWE water electrolysis device with HEA-ML‖Pt/C as electrodes at 500 mA cm^−2^ using the water displacement method. (c) Volumes of H_2_ generated and Faradaic efficiency of HEA-ML‖Pt/C in the AEMWE water electrolysis device at 500mA cm^−2^ under atmospheric pressure.

**Table S1** Summary of previously reported excellent OER catalysts in alkaline condition.

| **Catalyst** | **Overpotential (mV)**  **@ j (mA cm^-2^)** | **Tafel slop**  **(mV dec^−1^)** | **Electrolyte** | **References** |
| --- | --- | --- | --- | --- |
| **HEA-ML** | **230@10** | **39.4** | **1.0 M KOH** | **This work** |
| (CrMnFeCoNi) Sx | 295 @10 | 66 | 1.0 M KOH | ^[1]^ |
| (CrFeCoNi)_97_O_3_ | 196 @10 | 29 | 1.0 M KOH | ^[2]^ |
| ZnFeNiCuCoRu-O | 170 @10 | 56 | 1.0 M KOH | ^[3]^ |
| FeCoNiRu-450 | 243@100 | 45 | 1.0 M KOH | ^[4]^ |
| FeCoNiMnCr HEA-HEO/CNT | 261@10 | 42.2 | 0.1 M KOH | ^[5]^ |
| GaFeCoNiMo | 240@10 | 37.9 | 1.0 M KOH | ^[6]^ |
| HEO (CoFeNiCrMn) | 307@10 | 34.7 | 1.0 M KOH | ^[7]^ |
| HESA | 270@10 | 69.3 | 1.0 M KOH | ^[8]^ |
| Au_SA_-MnFeCoNiCu LDH | 213@10 | 27.5 | 1.0 M KOH | ^[9]^ |
| HEOs | 320@10 | 45 | 1.0 M KOH | ^[10]^ |
| La(CrMnFeCo_2_Ni)O_3_ | 325@10 | 51.2 | 1.0 M KOH | ^[11]^ |
| FeCoNiCuPd | 390@10 | 96 | 1.0 M KOH | ^[12]^ |
| FeCoNiMnMo | 279@10 | 56.1 | 1.0 M KOH | ^[13]^ |
| FeCoNiMnCu HEA | 280@10 | 59 | 1.0 M KOH | ^[14]^ |
| (Cr_0.2_Mn_0.2_Fe_0.2_Ni_0.2_Zn_0.2_)_3_O_4_ | 295@10 | 53.7 | 1.0 M KOH | ^[15]^ |
| Co-Fe-Ga-Ni-Zn | 370@10 | 71 | 1.0 M KOH | ^[16]^ |
| AlCrCuFeNi HEAs | 270@10 | 77.5 | 1.0 M KOH | ^[17]^ |
| S-HEO | 240@10 | 59 | 1.0 M KOH | ^[18]^ |
| FeCoNiMoWOx | 281@10 | 34.5 | 1.0 M KOH | ^[19]^ |

**Table S2** Summary of the OER performance and stability for the recently reported electrocatalysts in alkaline media.

| **Catalyst** | **Stability current density (mA cm^-2^)** | **Stability time (h)** | **Overpotential (mV)** | **References** |
| --- | --- | --- | --- | --- |
| **HEA-ML** | **1000** | **1533** | **230** | **This work** |
| Self-supporting catalysts | 500 | 1600 | 235 | ^[20]^ |
|  | 1000 | 100 | 200 | ^[21]^ |
|  | 1000 | 172 | 240 | ^[22]^ |
|  | 500 | 96 | 220 | ^[23]^ |
|  | 500 | 50 | 165 | ^[24]^ |
|  | 500 | 25 | 186 | ^[25]^ |
|  | 500 | 24 | 177 | ^[26]^ |
|  | 500 | 24 | 190 | ^[27]^ |
|  | 100 | 500 | 171 | ^[28]^ |
|  | 100 | 120 | 185 | ^[29]^ |
|  | 100 | 72 | 212 | ^[30]^ |
|  | 100 | 60 | 270 | ^[31]^ |
|  | 100 | 50 | 182 | ^[32]^ |
|  | 100 | 48 | 199 | ^[33]^ |
|  | 50 | 300 | 307 | ^[34]^ |
|  | 50 | 85 | 170 | ^[35]^ |
|  | 100 | 80 | 195 | ^[36]^ |
|  | 100 | 50 | 176 | ^[37]^ |
|  | 100 | 50 | 262 | ^[38]^ |
| Powder catalysts | 100 | 5 | 270 | ^[39]^ |
|  | 50 | 30 | 260 | ^[40]^ |
|  | 50 | 25 | 180 | ^[41]^ |
|  | 50 | 24 | 285 | ^[42]^ |
|  | 48 | 15 | 277 | ^[43]^ |
|  | 30 | 50 | 187 | ^[44]^ |
|  | 20 | 20 | 220 | ^[45]^ |
|  | 20 | 20 | 316 | ^[46]^ |
|  | 15 | 30 | 240 | ^[47]^ |
|  | 10 | 100 | 288 | ^[48]^ |
|  | 10 | 50 | 323 | ^[49]^ |
|  | 10 | 48 | 224 | ^[50]^ |
| Powder catalysts | 10 | 48 | 241 | ^[51]^ |
|  | 10 | 40 | 310 | ^[52]^ |
|  | 10 | 24 | 275 | ^[53]^ |
|  | 10 | 20 | 247 | ^[54]^ |
|  | 10 | 20 | 347 | ^[55]^ |
|  | 10 | 14 | 324 | ^[56]^ |
|  | 10 | 12 | 275 | ^[57]^ |
|  | 10 | 12 | 296 | ^[58]^ |
|  | 10 | 12 | 310 | ^[59]^ |
|  | 10 | 8 | 339 | ^[60]^ |
|  | 10 | 12 | 378 | ^[61]^ |
|  | 10 | 6 | 330 | ^[62]^ |

**Table S3** The electrochemical equivalent circuit fitting parameters for different catalysts.

|  | R_s_  (Ω cm^-2^) | R_1_  (Ω cm^-2^) | C_1_  (mF cm^-2^) | R_2_  (Ω cm^-2^) | Q_2_  (F s^(a-1)^) |
| --- | --- | --- | --- | --- | --- |
| HEA‖Pt/C | 0.164 | 1.19 | 30.17 | 3.899 | 0.098 |
| HEA-ML‖Pt/C | 0.153 | 0.274 | 149 | 1.092 | 0.038 |
| RuO_2_‖Pt/C | 0.206 | 0.705 | 5.3 | 2.603 | 0.037 |

**Table S4** Summary of previously reported excellent AEMWE electrodes.

| **Materials** | **Test condition** | **Potential (V)**  **@**  **j (mA cm^-2^)** | **References** |
| --- | --- | --- | --- |
| **HEA-ML**‖**Pt/C** | **30% KOH, 60℃** | **2.12@1000** | **This work** |
| NiCoTi‖NiCoTi | 1.0M KOH, 25℃ | 2 @170 | ^[63]^ |
| NiCo_2_O_4_‖NiCo_2_O_4_ | 1.0M KOH, 50℃ | 2 @400 | ^[64]^ |
| Ni_12_P_5_/Ni_3_(PO_4_)_2_-HS‖Ni_12_P_5_/Ni_3_(PO_4_)_2_-HS | 1.0M KOH, 50℃ | 1.87 @357.6 | ^[65]^ |
| CuNi@NiSe‖CuNi@NiSe | 1.0M KOH, 80℃ | 2.2@1000 | ^[66]^ |
| ac-NiCo(OH)_2_/NF‖ac-NiCo(OH)_2_/NF | 1.0M KOH, 25℃ | 1.92@100 | ^[67]^ |
| Ni-Fe‖Ni-Fe | 1.0M KOH, 60℃ | 2@1200 | ^[68]^ |
| Ni_3_S_4_@Ni(OH)_2_‖Ni_3_S_4_@Ni(OH)_2_ | 1.0M KOH, 25℃ | 1.95@1000 | ^[69]^ |
| WC_1-x_/Mo_2_C@CNF‖WC_1-x_/Mo_2_C@CNF | 1.0M KOH, 25℃ | 1.82@100 | ^[70]^ |
| NiCo–HCOO^–^‖Pt/C | 1.0M KOH, 25℃ | 2.01@400 | ^[71]^ |
| S-NiFe LDH‖Pt@S-NiFe LDH | 1.0M KOH, 25℃ | 2.5@500 | ^[72]^ |
| CoFeO_x_ ‖Pt black | 1.0M KOH, 25℃ | 2.15@500 | ^[73]^ |
| CoCrO_x_‖Pt/C | 1.0M KOH, 60℃ | 2.15@1.5 | ^[74]^ |
| CoO_x_‖Pt/C | 1.0M KOH, 60℃ | 2.4@1.4 | ^[74]^ |
| NiCrO_x_‖Pt/C | 1.0M KOH, 60℃ | 2.4@0.8 | ^[74]^ |
| IrO_2_‖PtC_60_ | 1.0M KOH, 60℃ | 2.39@2 | ^[75]^ |
| IrO_2_‖Pt/C | 1.0M KOH, 60℃ | 2.75@2 | ^[75]^ |
| IrO_2_‖Pt NCs | 1.0M KOH, 60℃ | 2.92@1.5 | ^[75]^ |

**Reference**

[1] M. Cui, C. Yang, B. Li, et al., “High-Entropy Metal Sulfide Nanoparticles Promise High-Performance Oxygen Evolution Reaction” *Adv. Energy Mater.* **11** (2021):2002887

[2] Z.-J. Chen, T. Zhang, X.-Y. Gao, et al., “Engineering Microdomains of Oxides in High-Entropy Alloy Electrodes toward Efficient Oxygen Evolution” *Adv. Mater.* **33** (2021):2101845

[3] K. Miao, W. Jiang, Z. Chen, et al., “Hollow-Structured and Polyhedron-Shaped High Entropy Oxide toward Highly Active and Robust Oxygen Evolution Reaction in a Full pH Range” *Adv. Mater.* **36** (2024):2308490

[4] K. Huang, J. Xia, Y. Lu, et al., “Self-Reconstructed Spinel Surface Structure Enabling the Long-Term Stable Hydrogen Evolution Reaction/Oxygen Evolution Reaction Efficiency of FeCoNiRu High-Entropy Alloyed Electrocatalyst” *Adv. Sci.* **10** (2023):2300094

[5] J. Hu, T. Guo, X. Zhong, et al., “In-situ Reconstruction of High-Entropy Heterostructure Catalysts for Stable Oxygen Evolution Electrocatalysis Under Industrial Conditions” *Adv. Mater.* **36** (2024):2310918

[6] J. Liang, J. Liu, H. Wang, et al., “Synthesis of Ultrathin High-Entropy Oxides with Phase Controllability” *J. Am. Chem. Soc.* **146** (2024):7118-7123

[7] J. Baek, M. D. Hossain, P. Mukherjee, et al., “Synergistic Effects of Mixing and Strain in High Entropy Spinel Oxides for Oxygen Evolution Reaction” *Nat. Commun.* **14** (2023):5936

[8] X. Lei, Q. Tang, Y. Zheng, et al., “High-Entropy Single-Atom Activated Carbon Catalysts for Sustainable Oxygen Electrocatalysis” *Nat. Sustain.* **6** (2023):816-826

[9] F. Wang, P. Zou, Y. Zhang, et al., “Activating Lattice Oxygen in High-entropy LDH for Robust and Durable Water Oxidation” *Nat. Commun.* **14** (2023):6019

[10] L. Tang, Y. Yang, H. Guo, et al., “High Configuration Entropy Activated Lattice Oxygen for O_2_ Formation on Perovskite Electrocatalyst” *Adv. Funct. Mater.* **32** (2022):2112157

[11] T. X. Nguyen, Y.-C. Liao, C.-C. Lin, et al., “Advanced High Entropy Perovskite Oxide Electrocatalyst for Oxygen Evolution Reaction” *Adv. Funct. Mater.* **31** (2021):2101632

[12] H. Li, H. Zhu, Q. Shen, et al., “A Novel Synergistic Confinement Strategy for Controlled Synthesis of High-Entropy Alloy Electrocatalysts” *Chem. Commun.* **57** (2021):2637-2640

[13] P. Li, X. Wan, J. Su, et al., “A Single-Phase FeCoNiMnMo High-Entropy Alloy Oxygen Evolution Anode Working in Alkaline Solution for over 1000 h” *ACS Catal.* **12** (2022):11667-11674

[14] K. Huang, D. Peng, Z. Yao, et al., “Cathodic Plasma Driven Self-Assembly of HEAs Dendrites by Pure Single FCC FeCoNiMnCu Nanoparticles as High Efficient Electrocatalysts for OER” *Chem. Eng. J.* **425** (2021):131533

[15] X. Yang, S. Liping, L. Qiang, et al., “Co-Prosperity of Electrocatalytic Activity and Stability in High Entropy Spinel (Cr_0.2_Mn_0.2_Fe_0.2_Ni_0.2_Zn_0.2_)_3_O_4_ for the Oxygen Evolution Reaction” *J. Mater. Chem. A* **10** (2022):17633-17641

[16] L. Sharma, N. K. Katiyar, A. Parui, et al., “Low-Cost High Entropy Alloy (HEA) for High-Efficiency Oxygen Evolution Reaction (OER)” *Nano Res.* **15** (2022):4799-4806

[17] L.-H. Liu, N. Li, M. Han, et al., “Scalable Synthesis of Nanoporous High Entropy Alloys for Electrocatalytic Oxygen Evolution” *Rare Met.* **41** (2022):125-131

[18] S. C. Karthikeyan, S. Ramakrishnan, S. Prabhakaran, et al., “Low-Cost Self-Reconstructed High Entropy Oxide as an Ultra-Durable OER Electrocatalyst for Anion Exchange Membrane Water Electrolyzer” *Small* **20** (2024):2402241

[19] L. Huang, L. Ma, J. Xu, et al., “Strong Electronic Interaction in High-Entropy Oxide Enhances Oxygen Evolution Reaction” *Inorg. Chem.* **63** (2024):12433-12444

[20] Z. Li, R. Wu, D. Duan, et al., “Empowering Multicomponent Alloys with Unique Nanostructure for Exceptional Oxygen Evolution Performance Through Self-Replenishment” *Joule* **8** (2024):2920 - 2937

[21] Z. Wang, G. Qian, T. Yu, et al., “Carbon Encapsulated FeWO_4_-Ni_3_S_2_ Nanosheets as a Highly Active Catalyst for Overall Water Splitting at Large Current Density” *Chem. Eng. J.* **434** (2022):134669

[22] G. Qian, G. Yu, J. Lu, et al., “Ultra-thin N-Doped-Graphene Encapsulated Ni Nanoparticles Coupled with MoO_2_ Nanosheets for Highly Efficient Water Splitting at Large Current Density” *J. Mater. Chem. A* **8** (2020):14545-14554

[23] Y. Xiao, T. Hu, X. Zhao, et al., “Thermo-Selenizing to Rationally Tune Surface Composition and Evolve Structure of Stainless Steel to Electrocatalytically Boost Oxygen Evolution Reaction” *Nano Energy* **75** (2020):104949

[24] X. Zou, Y. Liu, G.-D. Li, et al., “Ultrafast Formation of Amorphous Bimetallic Hydroxide Films on 3D Conductive Sulfide Nanoarrays for Large-Current-Density Oxygen Evolution Electrocatalysis” *Adv. Mater.* **29** (2017):1700404

[25] P. Zhai, Y. Zhang, Y. Wu, et al., “Engineering Active Sites on Hierarchical Transition Bimetal Oxides/Sulfides Heterostructure Array Enabling Robust Overall Water Splitting” *Nat. Commun.* **11** (2020):5462

[26] H. Zhou, F. Yu, J. Sun, et al., “Highly Active Catalyst Derived From a 3D Foam of Fe(PO_3_)_2_/Ni_2_P for Extremely Efficient Water Oxidation” *Proc. Natl. Acad. Sci. U.S.A.* **114** (2017):5607-5611

[27] P. Liu, B. Chen, C. Liang, et al., “Tip-Enhanced Electric Field: A New Mechanism Promoting Mass Transfer in Oxygen Evolution Reactions” *Adv. Mater.* **33** (2021):2007377

[28] Y. Wang, X. Li, M. Zhang, et al., “Highly Active and Durable Single-Atom Tungsten-Doped NiS_0.5_Se_0.5_ Nanosheet @ NiS_0.5_Se_0.5_ Nanorod Heterostructures for Water Splitting” *Adv. Mater.* **34** (2022):e2107053

[29] Y. Huang, L.-W. Jiang, B.-Y. Shi, et al., “Highly Efficient Oxygen Evolution Reaction Enabled by Phosphorus Doping of the Fe Electronic Structure in Iron–Nickel Selenide Nanosheets” *Adv. Sci.* **8** (2021):2101775

[30] Y. Yang, Y. Song, S. Mo, et al., “Efficient and Durable FeCoNi-(Oxy)hydroxide Anode: Stoichiometric Ration Regulated Morphology-, Defect- and Valence-Dependent Water Oxidation Performance” *Chem. Eng. J.* **417** (2021):127934

[31] Y. Wu, Z. Xie, Y. Li, et al., “In-Situ Self-Reconstruction of Ni–Fe–Al Hybrid Phosphides Nanosheet Arrays Enables Efficient Oxygen Evolution in Alkaline” *Int. J. Hydrog Energ.* **46** (2021):25070-25080

[32] L. Li, G. Zhang, B. Wang, et al., “Fe_2_O_3_/NiO Interface for the Electrochemical Oxygen Evolution in Seawater and Domestic Sewage” *ACS Appl. Mater. Interfaces* **13** (2021):37152-37161

[33] L. Yu, H. Zhou, J. Sun, et al., “Cu Nanowires Shelled with NiFe Layered Double Hydroxide Nanosheets as Bifunctional Electrocatalysts for Overall Water Splitting” *Energy Environ. Sci.* **10** (2017):1820-1827

[34] K. Wan, J. Luo, C. Zhou, et al., “Hierarchical Porous Ni_3_S_4_ with Enriched High-Valence Ni Sites as a Robust Electrocatalyst for Efficient Oxygen Evolution Reaction” *Adv. Funct. Mater.* **29** (2019):1900315

[35] Y.-N. Zhou, M.-X. Li, S.-Y. Dou, et al., “Promoting Oxygen Evolution by Deep Reconstruction via Dynamic Migration of Fluorine Anions” *ACS Appl. Mater. Interfaces* **13** (2021):34438-34446

[36] F. Nur Indah Sari, S. Abdillah, J.-M. Ting, “FeOOH-Containing Hydrated Layered Iron Vanadate Electrocatalyst for Superior Oxygen Evolution Reaction and Efficient Water Splitting” *Chem. Eng. J.* **416** (2021):129165

[37] C. Wang, P. Zhai, M. Xia, et al., “Engineering Lattice Oxygen Activation of Iridium Clusters Stabilized on Amorphous Bimetal Borides Array for Oxygen Evolution Reaction” *Angew. Chem. Int. Ed.* **60** (2021):27126-27134

[38] S. L. Zhang, B. Y. Guan, X. F. Lu, et al., “Metal Atom-Doped Co_3_O_4_­ Hierarchical Nanoplates for Electrocatalytic Oxygen Evolution” *Adv. Mater.* **32** (2020):2002235

[39] C. H. Ahn, N. G. Deshpande, H. S. Lee, et al., “Atomically Controllable In-Situ Electrochemical Treatment of Metal-Organic-Framework-Derived Cobalt-Embedded Carbon Composites for Highly Efficient Electrocatalytic Oxygen Evolution” *Appl. Surf. Sci.* **554** (2021):149651

[40] D. C. Nguyen, T. L. Luyen Doan, S. Prabhakaran, et al., “Hierarchical Co and Nb Dual-Doped MoS_2_ Nanosheets Shelled Micro-TiO_2_ Hollow Spheres as Effective Multifunctional Electrocatalysts for HER, OER, and ORR” *Nano Energy* **82** (2021):105750

[41] J. Lee, A. Kumar, T. Yang, et al., “Stabilizing the OOH* Intermediate via Pre-Adsorbed Surface Oxygen of a Single Ru Atom-Bimetallic Alloy for Ultralow Overpotential Oxygen Generation” *Energy Environ. Sci.* **13** (2020):5152-5164

[42] X. Zheng, P. Cui, Y. Qian, et al., “Multifunctional Active-Center-Transferable Platinum/Lithium Cobalt Oxide Heterostructured Electrocatalysts towards Superior Water Splitting” *Angew. Chem. Int. Ed.* **59** (2020):14533-14540

[43] L. Fan, B. Zhang, B. J. J. Timmer, et al., “Promoting the Fe(VI) Active Species Generation by Structural and Electronic Modulation of Efficient Iron Oxide Based Water Oxidation Catalyst without Ni or Co” *Nano Energy* **72** (2020):104656

[44] Z. Li, X. Zhang, Y. Kang, et al., “Interface Engineering of Co-LDH@MOF Heterojunction in Highly Stable and Efficient Oxygen Evolution Reaction” *Adv. Sci.* **8** (2021):2002631

[45] D. Guo, Z. Zeng, Z. Wan, et al., “A CoN-based OER Electrocatalyst Capable in Neutral Medium: Atomic Layer Deposition as Rational Strategy for Fabrication” *Adv. Funct. Mater.* **31** (2021):2101324

[46] G. E. Ayom, M. D. Khan, G. B. Shombe, et al., “Triphenylphosphine-Assisted Transformation of NiS to Ni_2_P through a Solvent-Less Pyrolysis Route: Synthesis and Electrocatalytic Performance” *Inorg. Chem.* **60** (2021):11374-11384

[47] H. Xu, D. Song, J. Li, et al., “Chlorine-Assisted Synthesis of CuCo_2_S_4_@(Cu,Co)_2_Cl(OH)_3_ Heterostructures with an Efficient Nanointerface for Electrocatalytic Oxygen Evolution” *J. Colloid Interf. Sci.* **601** (2021):437-445

[48] M. Li, Y. Zhu, H. Wang, et al., “Ni Strongly Coupled with Mo_2_C Encapsulated in Nitrogen-Doped Carbon Nanofibers as Robust Bifunctional Catalyst for Overall Water Splitting” *Adv. Energy Mater.* **9** (2019):1803185

[49] C. Yang, M. Cui, N. Li, et al., “In Situ Iron Coating on Nanocatalysts for Efficient and Durable Oxygen Evolution Reaction” *Nano Energy* **63** (2019):103855

[50] G. Zhao, P. Li, N. Cheng, et al., “An Ir/Ni(OH)_2_ Heterostructured Electrocatalyst for the Oxygen Evolution Reaction: Breaking the Scaling Relation, Stabilizing Iridium(V), and Beyond” *Adv. Mater.* **32** (2020):2000872

[51] X. Hou, Z. Han, X. Xu, et al., “Controllable Amorphization Engineering on Bimetallic Metal–Organic Frameworks for Ultrafast Oxygen Evolution Reaction” *Chem. Eng. J.* **418** (2021):129330

[52] M.-H. Fang, H.-P. Hsueh, T. Vasudevan, et al., “Dual-Emission Eu-Doped Ca_2−x_Sr_x_PN_3_ Nitridophosphate Phosphors Prepared by Hot Isostatic Press” *J. Mater. Chem. C* **9** (2021):8158-8162

[53] S. Javaid, X. Xu, W. Chen, et al., “Ni^2+^/Co^2+^ Doped Au-Fe7S8 Nanoplatelets with Exceptionally High Oxygen Evolution Reaction Activity” *Nano Energy* **89** (2021):106463

[54] C. Rong, X. Shen, Y. Wang, et al., “Electronic Structure Engineering of Single-Atom Ru Sites via Co–N_4_ Sites for Bifunctional pH-Universal Water Splitting” *Adv. Mater.* **34** (2022):2110103

[55] Q. Liang, H. Jin, Z. Wang, et al., “Metal-Organic Frameworks Derived Reverse-Encapsulation Co-NC@Mo_2_C Complex for Efficient Overall Water Splitting” *Nano Energy* **57** (2019):746-752

[56] M.-Y. Ye, S. Li, X. Zhao, et al., “Cobalt-Exchanged Poly(Heptazine Imides) as Transition Metal–N Electrocatalysts for the Oxygen Evolution Reaction” *Adv. Mater.* **32** (2020):1903942

[57] B. Tang, X. Yang, Z. Kang, et al., “Crystallized RuTe_2_ as Unexpected Bifunctional Catalyst for Overall Water Splitting” *Appl. Catal. B: Environ.* **278** (2020):119281

[58] F. Luo, L. Guo, Y. Xie, et al., “Iridium Nanorods as a Robust and Stable Bifunctional Electrocatalyst for pH-Universal Water Splitting” *Appl. Catal. B: Environ.* **279** (2020):119394

[59] M. Chauhan, K. P. Reddy, C. S. Gopinath, et al., “Copper Cobalt Sulfide Nanosheets Realizing a Promising Electrocatalytic Oxygen Evolution Reaction” *ACS Catal.* **7** (2017):5871-5879

[60] C. Hu, H. Jin, B. Liu, et al., “Propagating Fe-N_4_ Active Sites with Vitamin C to Efficiently Drive Oxygen Electrocatalysis” *Nano Energy* **82** (2021):105714

[61] D. Wang, Y. Wang, Z. Fu, et al., “Cobalt–Nickel Phosphate Composites for the All-Phosphate Asymmetric Supercapacitor and Oxygen Evolution Reaction” *ACS Appl. Mater. Interfaces* **13** (2021):34507-34517

[62] X. Han, C. Yu, S. Zhou, et al., “Ultrasensitive Iron-Triggered Nanosized Fe–CoOOH Integrated with Graphene for Highly Efficient Oxygen Evolution” *Adv. Energy Mater.* **7** (2017):1602148

[63] P. Ganesan, A. Sivanantham, S. Shanmugam, “Nanostructured Nickel–Cobalt–Titanium Alloy Grown on Titanium Substrate as Efficient Electrocatalyst for Alkaline Water Electrolysis” *ACS Appl. Mater. Interfaces* **9** (2017):12416-12426

[64] M. Bhushan, M. Mani, A. K. Singh, et al., “Self-Standing Polyaniline Membrane Containing Quaternary Ammonium Groups Loaded with Hollow Spherical NiCo_2_O_4_ Electrocatalyst for Alkaline Water Electrolyser” *J. Mater. Chem. A* **8** (2020):17089-17097

[65] J. Chang, Q. Lv, G. Li, et al., “Core-Shell Structured Ni_12_P_5_/Ni_3_(PO_4_)_2_ Hollow Spheres as Difunctional and Efficient Electrocatalysts for Overall Water Electrolysis” *Appl. Catal. B: Environ.* **204** (2017):486-496

[66] D. Cao, J. Shao, Y. Cui, et al., “Interfacial Engineering of Copper–Nickel Selenide Nanodendrites for Enhanced Overall Water Splitting in Alkali Condition” *Small* **19** (2023):2301613

[67] S. Ju, Y. Liu, M. Pei, et al., “Amorphization-Induced Abundant Coordinatively Unsaturated Ni Active Sites in NiCo(OH)_2_ for Boosting Catalytic OER and HER Activities at High Current Densities for Water-Electrolysis” *J. Colloid Interf. Sci.* **653** (2024):1704-1714

[68] N. Chen, S. Y. Paek, J. Y. Lee, et al., “High-Performance Anion Exchange Membrane Water Electrolyzers with a Current Density of 7.68 A cm^−2^ and a Durability of 1000 Hours” *Energy Environ. Sci.* **14** (2021):6338-6348

[69] H. Chai, X. Ma, Y. Dang, et al., “Triple Roles of Ni(OH)_2_ Promoting the Electrocatalytic Activity and Stability of Ni_3_S_4_@Ni(OH)_2_ in Anion Exchange Membrane Water Electrolyzers” *J. Colloid Interf. Sci.* **654** (2024):66-75

[70] W. Zhang, L. Yang, Z. Li, et al., “Regulating Hydrogen/Oxygen Species Adsorption via Built-in Electric Field -Driven Electron Transfer Behavior at the Heterointerface for Efficient Water Splitting” *Angew. Chem. Int. Ed.* **63** (2024):e202400888

[71] X. Wang, J. Song, J. Ma, et al., “Surface-Bound Formate Oxyanions Destabilize Hydration Layers to Pave OH^–^ Transport Pathways for Oxygen Evolution” *ACS Catal.* **14** (2024):10871-10881

[72] H. Lei, Q. Wan, S. Tan, et al., “Pt-Quantum-Dot-Modified Sulfur-Doped NiFe Layered Double Hydroxide for High-Current-Density Alkaline Water Splitting at Industrial Temperature” *Adv. Mater.* **35** (2023):2208209

[73] D. Xu, M. B. Stevens, M. R. Cosby, et al., “Earth-Abundant Oxygen Electrocatalysts for Alkaline Anion-Exchange-Membrane Water Electrolysis: Effects of Catalyst Conductivity and Comparison with Performance in Three-Electrode Cells” *ACS Catal.* **9** (2019):7-15

[74] S. Li, T. Liu, W. Zhang, et al., “Highly Efficient Anion Exchange Membrane Water Electrolyzers via Chromium-Doped Amorphous Electrocatalysts” *Nat. Commun.* **15** (2024):3416

[75] J. Chen, M. Aliasgar, F. B. Zamudio, et al., “Diversity of Platinum-Sites at Platinum/Fullerene Interface Accelerates Alkaline Hydrogen Evolution” *Nat. Commun.* **14** (2023):1711
